# Poly(3,4-ethylenedioxythiophene) (PEDOT) Derivatives: Innovative Conductive Polymers for Bioelectronics

**DOI:** 10.3390/polym9080354

**Published:** 2017-08-11

**Authors:** Daniele Mantione, Isabel del Agua, Ana Sanchez-Sanchez, David Mecerreyes

**Affiliations:** 1 Polymat University of the Basque Country UPV/EHU, Joxe Mari Korta Center, Avda. Tolosa 72, 20018 Donostia-San Sebastian, Spain; daniele.mantione@ehu.es (D.M.); isabel.delagua@ehu.es (I.d.A.); 2 Department of Bioelectronics, Ecole Nationale Supérieure des Mines, CMP-EMSE, MOC, 13541 Gardanne, France; 3 Ikerbasque, Basque Foundation for Science, E-48011 Bilbao, Spain

**Keywords:** PEDOT, conducting polymers, biopolymers, bioelectronics

## Abstract

Poly(3,4-ethylenedioxythiophene)s are the conducting polymers (CP) with the biggest prospects in the field of bioelectronics due to their combination of characteristics (conductivity, stability, transparency and biocompatibility). The gold standard material is the commercially available poly(3,4-ethylenedioxythiophene):poly(styrene sulfonate) (PEDOT:PSS). However, in order to well connect the two fields of biology and electronics, PEDOT:PSS presents some limitations associated with its low (bio)functionality. In this review, we provide an insight into the synthesis and applications of innovative poly(ethylenedioxythiophene)-type materials for bioelectronics. First, we present a detailed analysis of the different synthetic routes to (bio)functional dioxythiophene monomer/polymer derivatives. Second, we focus on the preparation of PEDOT dispersions using different biopolymers and biomolecules as dopants and stabilizers. To finish, we review the applications of innovative PEDOT-type materials such as biocompatible conducting polymer layers, conducting hydrogels, biosensors, selective detachment of cells, scaffolds for tissue engineering, electrodes for electrophysiology, implantable electrodes, stimulation of neuronal cells or pan-bio electronics.

## 1. Introduction

Electrically conductive polymers are of great interest in the field of bioelectronics as materials that can improve the interface between electronics and biology. Among different conducting polymers, poly(3,4-ethylenedioxythiophene):poly(styrene sulfonate) (PEDOT:PSS) is the most promising due to its high conductivity, easy processing and commercial availability [1,2]. PEDOT:PSS is commercialized in the form of an aqueous dispersion which can be processed in the form of thin films by spin-coating and solvent-casting methods. PEDOT:PSS is widely used in the field of organic electronics as transparent conductive oxides (TCO), and as a hole-conducting layer or electrochromic layer in a variety of devices from organic light-emitting diodes (OLEDs) and organic photovoltaic devices (OPVs) [3,4] to electrochromics [5,6]. In recent years, PEDOT:PSS has also been widely applied in bioelectronic devices for applications as electrodes for electrophysiology, a variety of biosensors, organic electrochemical transistors (OECTs) and small bioelectrode coatings [6,7,8,9,10,11,12,13,14,15]. However, in order to connect well the two fields of biology and electronics, PEDOT:PSS presents some limitations, mostly due to its low biofunctionality and biocompatibility.

In most bioelectronic applications, the fine tuning of the interface of conducting polymers and biological molecules or tissues/organisms is a crucial parameter. As one illustrative example, the biotic/abiotic interface for interfacing with live cells can be improved by the incorporation of biological molecules such as nucleotides or proteins for functionality, e.g., for sensing. In this way, the biofunctionalized conductive polymer can enhance their ultimate properties such as biocompatibility and adhesion, and could help to reduce the inflammatory response of a device in living tissue. The two components of PEDOT:PSS are limited, due on the one hand to the lack of functionality of PEDOT and on the other hand to the low biocompatibility of PSS. To overcome these limitations, much effort has been devoted to design innovative poly(dioxythiophene) polymers containing different functional groups for improved biocompatibility [16,17].

**Figure 1:**
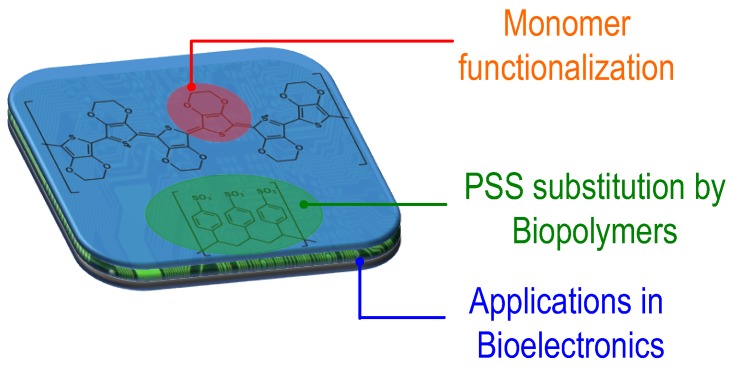
Graphical representation of alternative materials to PEDOT:PSS described in this review.

In this review, we summarize recent advances in the design of innovative conducting polydioxythiophene (PEDOT poly(3,4-ethylenedioxythiophene) or ProDOT poly(3,4-Propylenedioxythiophene))-type materials for bioelectronics (Figure 1). The broadest used material today is the commercially available poly(3,4-ethylenedioxythiophene):poly(styrene sulfonate) (PEDOT:PSS) aqueous dispersion which has two main components: PEDOT and PSS. First, we focus our efforts on discussing the different synthetic routes to modify the first component PEDOT. Thus, we will review the different functional EDOT and ProDOT monomers and their derivatives that have been reported in the scientific literature. Next, we review the different trials to substitute PSS, the second component of the material. Here, we review the synthesis of PEDOT:biopolymer aqueous dispersions. Finally, we discuss how these innovative functional PEDOT materials are widening the scope of the applications of conducting polymers in bioelectronics.

## 2. Synthesis of Functional Ethylenedioxythiophene (EDOT-ProDOT) Monomers

**Figure 2:**
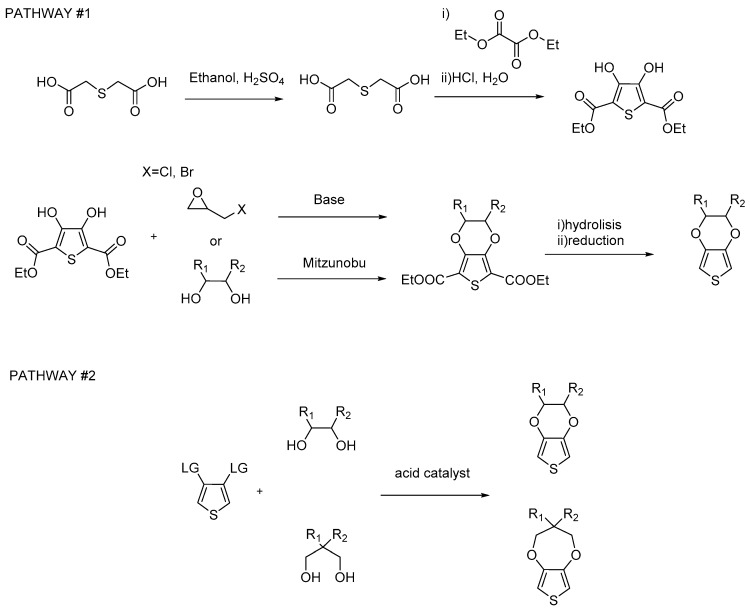
The two most used synthetic pathways for the synthesis of EDOT and ProDOT monomers.

There are two main different methods to synthesize functional 3,4-ethylenedioxothiophene monomers. One synthetic route involves the creation of the thiophene and dioxolane derivatives (Figure 2 pathway #1). In the second synthetic route, the starting material is a 3,4-substituted thiophene ring, for example 3,4-dimethoxythiophene or 3,4-dibromothiophene (Figure 2 pathway #2). The macroscopic difference between the two synthetic routes is the number of steps to reach the final product: in the case of pathway #1, more steps are needed, but the price of the starting material for pathway #2 is about 10 to 100 times more expensive than for pathway #1. It is worth mentioning that pathway #1 is the oldest synthetic route and has only been employed for the synthesis of 2-hydroxymethyl-EDOT (EDOT-CH_2_OH) and carboxy-EDOT (EDOT-COOH) [13,16]. In the case of EDOT-CH_2_OH following pathway #2, the reaction with glycerol tends to yield a mixture of EDOT and ProDOT that is difficult to separate. In the case of EDOT-COOH, PCC (pyridinium chlorochromate) is used in an oxidative step and the groups in position 2 and 5 on the thiophene ring protect the molecule from further polymerization when pathway #1 is used [13]. For the ProDOT derivatives, pathway #2 is the only route used in the literature, whereby 1,3-diol derivatives are used to create the dioxepane ring.

**Figure 3:**
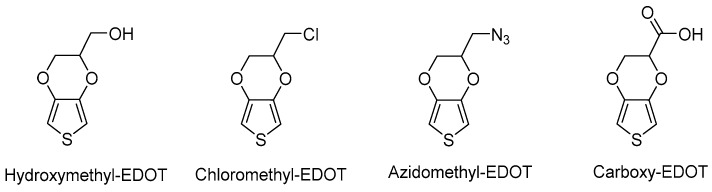
The most used functional EDOT derivatives.

The most representative functional EDOT monomers are shown in Figure 3. These molecules, which can be synthesized using both pathways previously described, include hydroxymethyl-EDOT, chloromethyl-EDOT, azidomethyl-EDOT and carboxy-EDOT. These functional monomers have been used in order to obtain functional PEDOT polymers including hydroxyl, halide or carboxylic acid functionalities. Furthermore, as will be shown in the following paragraphs, they have been widely used as basic reagents to synthesize a large variety of EDOT monomers with other functionalities.

**Figure 4:**
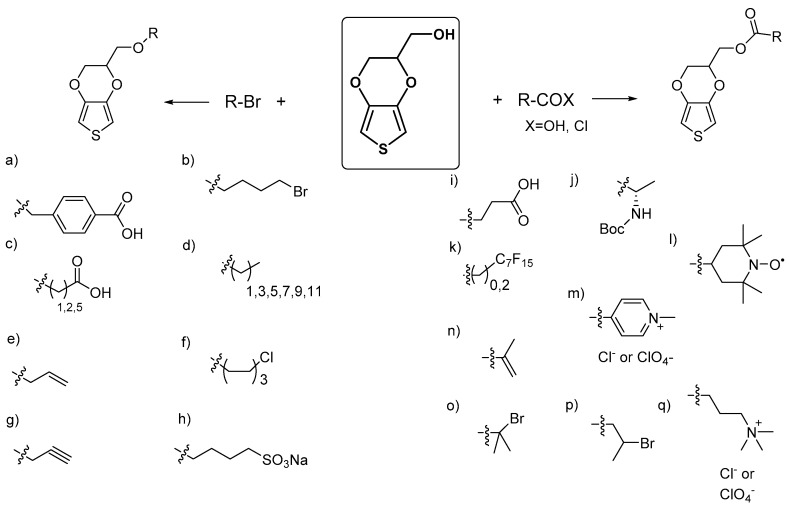
The most representative functional EDOT monomers synthesized from hydroxymethyl-EDOT. On the left side, the synthetic pathway is shown using alkylbromides to connect an (**a**) aromatic acid [18], (**b**) aliphatic bromine [19], (**c**) aliphatic acid [20,21,22,23], (**d**) aliphatic chains [24,25], (**e**) alkene [26], (**f**) halogenated aliphatic [27,28], (**g**) alkyne [29,30], and (**h**) aliphatic sulfonate [31] moieties. On the right side, the pathway is shown using carboxylic acid derivatives to connect an (**i**) aliphatic acid [23], (**j**) protected amino acid [32], (**k**) fluorinated aliphatic chain [24,33,34], (**l**) TEMPO ((2,2,6,6-Tetramethylpiperidin-1-yl)oxyl) [35], (**m**) pyridinium [36], (**n**) alkene [37], (**o**) tertiary bromide [38], (**p**) secondary bromide [39], and (**q**) quaternized ammonium salt moieties [40].

Hydroxymethyl-EDOT is the most widely studied and used EDOT derivative which makes the molecule commercially available [16,17,31,41,42,43]. As a monomer, it offers the advantage of improving the solubility of EDOT in water, and it can be co-polymerized giving PEDOTs of high electrical conductivity. As a synthetic brick, it opens the way to several functionalities by chemically modifying the hydroxyl group. Two main strategies have been exploited to obtain functional EDOT monomers. One involves the etherification of the hydroxyl group by nucleophilic substitution using an alkylbromide group, and the other uses an esterification with a carboxylic acid derivative, as shown in Figure 4. In the first case, new EDOT molecules including aromatic and aliphatic carboxylic acid groups, alkene, alkyne, alkyl halide and sulfonate groups have been synthesized (Figure 4, left side). In the second case, EDOT monomers including activated alkenes and halides, pyridinium, alkylammonium, protected amines, naphthalene diimide and nitroxide functionalities have been reported, as shown in Figure 4 on the right side.

**Figure 5:**
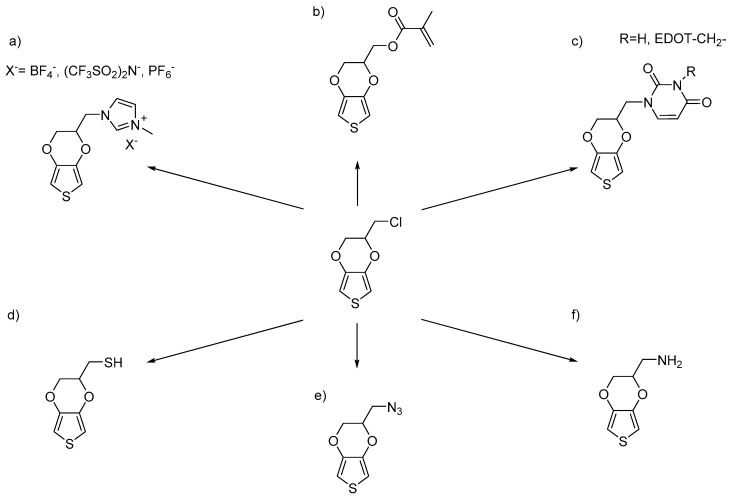
Most representative functional EDOT monomers synthesized from chloromethyl-EDOT. Substituted with: (**a**) imidazolium salts [44,45], (**b**) methacrylate [46], (**c**) pyrimidine bases [47], (**d**) thiole [26,48], (**e**) azide [49,50,51,52,53], and (**f**) amino moieties [54,55].

**Figure 6:**
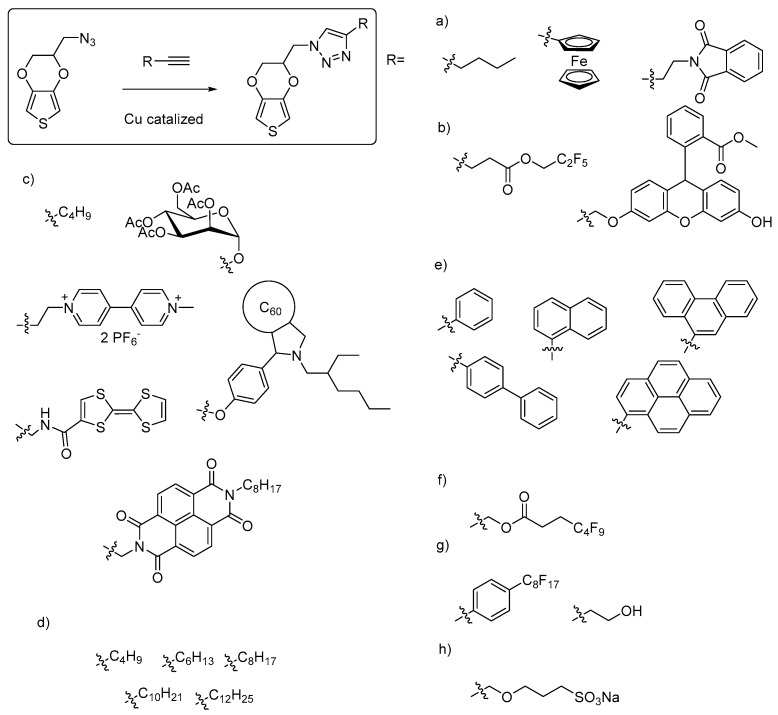
Most representative functional EDOT monomers synthesized from azidomethyl-EDOT using click chemistry: (**a**) aliphatic, ferrocene phtalamide [50,52], (**b**) fluorinated ester, substituted xantene [51], (**c**) sugar, bipyridine, naphtalenediimide, substituted fullerene [63], (**d**) plain aliphatic chains [49,64], (**e**) plain aromatic groups [65], (**f**) fluorinated ester [64], (**g**) fluorinated aromatic, primary alcohol [53], and (**h**) sulfonate salt, have all been connected to the EDOT scaffold via a triazole spacer [66].

Another important reagent is chloromethyl-EDOT, first synthesized by Segura et al. [56,57] and further optimized by many other research groups [18,49,50,58,59,60,61,62]. Using this molecule, functionalities can be incorporated by using the chlorine atom as a leaving group to form ether, amine or ester linkages. Using this monomer, new EDOT derivatives including imidazolium, thiol, azide, primary amine, uracil and methacrylate functionalities have been synthesized (Figure 5). Similar chemistry can be applied to the bromomethyl EDOT derivative. Among the EDOT molecules produced by this route, azidomethyl-EDOT is a powerful scaffold used in the Cu-catalysed cycloadditions of click chemistry in combination with terminal alkynes (Figure 6). Using azidomethyl-EDOT, further molecules having a triazole spacer have been synthesized containing different functionalities such as alkyl, aromatic, fluorinated, hydroxyl, sulfonate, ferrocenium, viologen, tetrathiafulvalene, naphthalene diimide, fullerene or saccharide moieties.

**Figure 7:**
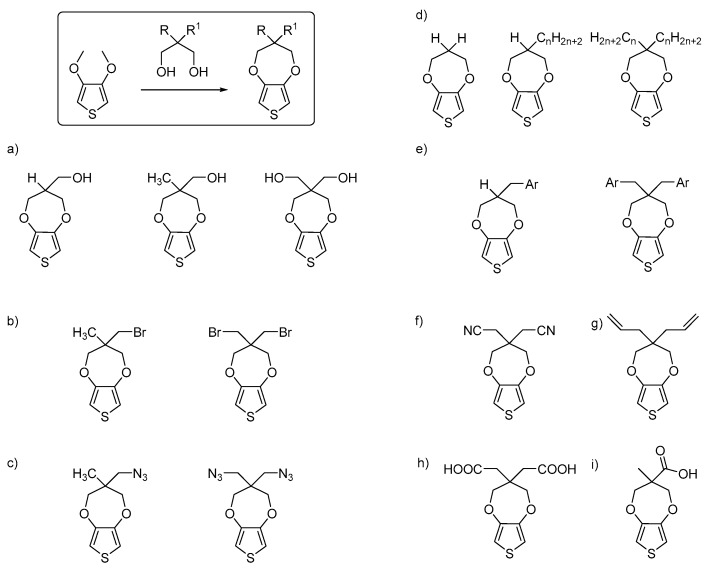
ProDOT main synthetic pathway and derivatives: (**a**) primary alcohols and diol [24,81,82,83,84,85,86,87,88], (**b**) primary mono- and di-bromide [71,72,73,74,75,76,77], (**c**) mono- and di-azide [30,71,72,73,74,75,76,77,80], (**d**) mono- and di-aliphatic chains [58,81,89,90,91,92,93,94,95,96,97,98,99], (**e**) mono- and di-aromatic groups [67,68,69,70], (**f**) di-cyano [78], (**g**) di-alkene [79], (**h**) di-carboxylic acid [78], (**i**) carboxylic acid [100].

The closest brother of the EDOT molecule is ProDOT which has a propylenedioxy ring instead of a ethylenedioxy ring attached to the thiophene in the 3,4-position. ProDOT was originally considered a side product in the synthesis. In fact, ProDOT is both easier to obtain and is more stable than EDOT, and even if ProDOT’s electrical conductivity is less than EDOT, it can still be used in applications such as OPV, OECT, tissue engineering and more, after doping or coupling with EDOT. The synthesis of the ProDOT derivatives in literature has been performed mostly only with pathway #2 as previously described. As shown in Figure 7, a 1,3-diol was reacted with a 3,4-dimethoxythiophene, leading to the substituted dioxepine ring. Many different diols have been used, leading to a wide library of functional ProDOT monomers. Summarizing them, monomers—including aliphatic moieties, perfluorinated aliphatic chains, hydroxyl groups, aromatic groups such as substituted and unsubstituted benzenes or naphthalene—have been inserted, principally to enhance optical properties [67,68,69,70]. ProDOTs functionalized with alkyl bromide [71,72,73,74,75,76,77], cyano [78], allyl [79], azide [30,71,72,73,74,75,76,77,80], or di- and mono-carboxylic acid [78] groups have been obtained as starting materials for further monomer and polymer functionalizations, as detailed in Figure 7.

## 3. Innovative PEDOT Biopolymer Aqueous Dispersions

Today, it is well known that EDOT/ProDOT monomers can be polymerized using different methods such as electrochemical polymerization, vapor phase polymerization (VPP) or chemical oxidative polymerization. Electrochemical and VPP polymerization methods usually give polymer films with very good properties such as surface quality, high conductivity and very stable redox chemistry. However, for large-scale applications, chemical polymerization is the preferred route due to its easy scale-up. For instance, this is the method used to produce industrially the PEDOT:PSS dispersions that are commercially available. PEDOT:PSS is currently being used in the area of bioelectronics due to its low toxicity with several cell types including endothelial, epithelial, fibroblast, macrophage, and most importantly human neuronal cell lines in vitro [101,102,103,104]. However, regardless of how promising this material has proven to be, the long-term effects such as PEDOT chains degradation and possible release of acidic PSS degradation products remains a potential issue [105].

A demonstrated strategy to enhance the biocompatibility and reduce the cytotoxicity of conducting polymers like PEDOT is the use of biomolecules as dopants. Thus, the incorporation of biopolymers could be the way to overcome the limitations of PEDOT:PSS dispersions for specific applications. Although PEDOT:PSS has proven to be an appropriate material for cell culture [102,106], the aim is to provide an environment that stimulates and persuades cell growth [105]. The first attempts to synthetize PEDOT doped by biopolymers was pioneered by Inganäs et al. by the electropolymerization of EDOT in the presence of biomolecules (heparin, hyaluronic acid, fibrinogen, gellan gum, carboxymethyl cellulose, xanthan gum, pectin and gellan gum) [107,108,109] showing their potential due to their non-toxicity [37] and finding applications as electrode interfaces for cell recordings [105,108]. Inspired by these polymer composites formed by PEDOT, different water-based PEDOT:biopolymer dispersions have been more recently synthetized by different groups using chemical polymerization.

**Figure 8:**
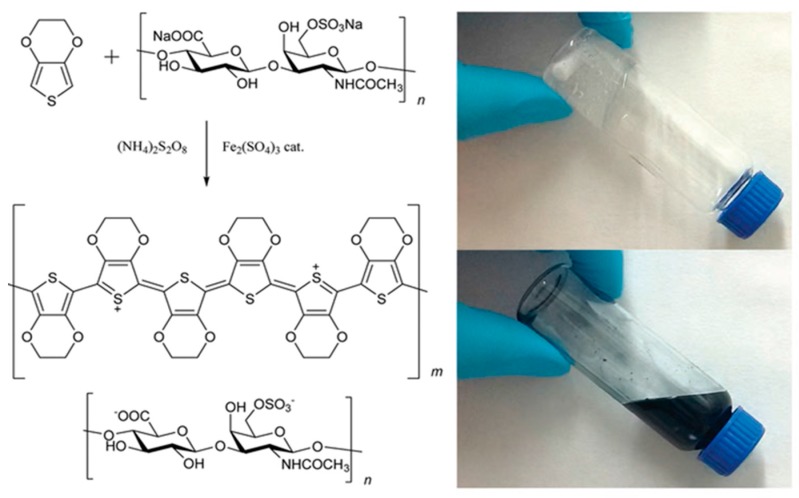
Synthetic route to PEDOT:Hyaluronic acid aqueous dispersions [113].

PEDOT:biopolymer aqueous dispersions synthesized by chemical polymerization have been reported using DNA [110], sulfated cellulose [111], dextran sulfonate [112], hyaluronic acid, heparin, chondroitin sulfate [113], pectin [114] and guar gum [115]. The synthesis of all of them is very similar and it can be exemplified in Figure 8 for the case of PEDOT:hyalunoric acid dispersions. A typical PEDOT:biopolymer dispersion is synthesized by chemical oxidative polymerization of the EDOT monomer using an oxidant in the presence of a biopolymer as a stabilizer and dopant. However, some parameters vary from one synthesis to another, and these include the PEDOT:biopolymer ratio, reaction temperature, concentration, time and oxidant used. In a typical experimental set-up, these biomolecules and EDOT are firstly dissolved in water; once dissolved, the oxidant is added to the solution. This oxidant can be ammoniumpersulfate ((NH_4_)_2_S_2_O_8_), potassium persulfate (K_2_S_2_O_8_), iron (III) chloride (FeCl_3_) or iron (III) *p*-toluenesulfonate ((CH_3_C_6_H_4_SO_3_)_3_Fe). A catalyst is often employed such as iron (II) sulfate (Fe_2_(SO_4_)_3_) to accelerate the reaction kinetics. Once the reaction is complete, the dispersions are purified by ion exchange, filtered and/or dialyzed.

The PEDOT:biopolymer dispersions have a macroscopic aspect similar to PEDOT:PSS dispersions. As can be seen in the picture of Figure 8, dark blue dispersions are obtained. The dispersions are formed by PEDOT particles of sizes between 100–500 nm stabilized by the biopolymer. Particle size and morphology can be studied by UV-spectroscopy, light-scattering, scanning electron microscopy (SEM), and transmission electron microscopy (TEM). Similarly to PEDOT:PSS, the PEDOT:biopolymer dispersions can be processed in the form of thin films or the solution formulated to be inkjet printed, extrusion printed and spray coated. The electrochemical properties of the PEDOT:biopolymer films, for instance PEDOT:dextran sulfate or PEDOT:DNA present similar features to PEDOT:PSS. Furthermore, the electrical conductivity of drop-casted or spin-coated films presents similar values to pristine PEDOT:PSS without further treatments of between 10^−1^–10 S·cm^−1^.

**Figure 9:**
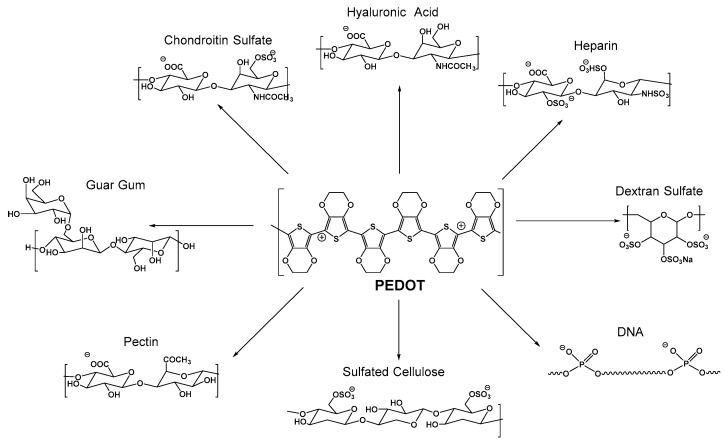
PEDOT:biopolymer dispersions employing hyaluronic acid, heparin, chondroitin sulfate [113], dextran sulfate [112], DNA [110], sulfated cellulose [111], pectin [114], and guar gum [115].

Using this method, different PEDOT:biopolymer dispersions have been prepared as shown in Figure 9 and summarized in Table 1. The advantages of each PEDOT:biopolymer material in comparison to PEDOT:PSS are discussed here. PEDOT:dextran sulfate presents two advantages. Firstly, it does not interfere with cell growth of L-929 cells in media in contrast with decreased cell numbers in culture when PEDOT:PSS is tested [112]. Secondly, PEDOT:dextran sulfate was absorbed into the PC12 cells while PEDOT:PSS was not. In the case of the PEDOT:DNA complex, the main advantages are its higher conductivity with respect to PEDOT:PSS and its non-acidic nature [110]. In the case of PEDOT:sulfate cellulose, it shows a higher conductivity than PEDOT:PSS which has been attributed to a higher proportion of PEDOT chains of quinoid structure than in PEDOT:PSS. In the case of PEDOT:glycosaminoglycans, they provide functional support in neuroregenerative processes and in the case of chondroitin sulfate, additional protection in oxidative milieu [113].

In particular, PEDOT:biopolymers have been biologically tested by cell proliferation of a fibroblast cell line (L-929 cells) in the cases of dextran, heparin, hyaluronic acid and chondroitin sulfate. The biological study was more extended in the case of the glycosaminoglycans [113], including cytotoxicity assays, SH-SY5Y differentiation studies and immunocytochemistry, and intracellular calcium measurements. In these studies, there are many biological findings. Hyaluronic acid, heparin and chondroitin sulfate do not interfere with physiological functions. They are more supportive for neuroregenerative processes compared to PEDOT:PSS and provide functional support. Moreover, chondroitin sulfate was found to have a neuroprotective effect against H_2_O_2_-induced cell death on SH-SY5Y cells. In the case of PEDOT:dextran sulfate, the studies showed higher L-929 cell line proliferation than PEDOT:PSS.

Although there are thorough biocompatibility studies of these dispersions as mentioned above, little is known about their long-term effects when implanted. The longest cytotoxic test performed so far had a duration of 96 h in the case of PEDOT:dextran sulfate. Although promising because of their satisfactory findings, these dispersions should undergo deeper study regarding their stability and possible long-term effects when in contact with living tissue.

PEDOT:biopolymer dispersions have great potential due to their improved biocompatibility, combined features and bio-based nature. The methods to increase the conductivity of these materials remains an unaddressed issue with respect to the well-known methods used to increase the conductivity of PEDOT:PSS. This relatively low conductivity of PEDOT:biomolecules limits their applicability in some applications such as bioelectrodes [105]. The study of the effect of solvents or secondary dopants should be addressed. For instance, this effect was studied in the case of PEDOT:dextran sulfate, where ethylene glycol was added to the dispersion achieving higher conductivities (286% increase in conductivity reaching 20 S·cm^−1^). As polar organic compounds, ionic liquids and acids are able to induce phase separation in between PEDOT and PSS chains, PEDOT chains become more linearly oriented and more interconnected [116,117,118]. Treatments like these could be studied to induce the same consequences in PEDOT chains when biomolecules are used as dopants. Moreover, in order to extend their use, PEDOT:biomolecule dispersions should be applied and processed using different printing and coating techniques. For this reason, more applicability and synthesis studies should be performed to extend their use. If these limitations are encountered, PEDOT:biomolecule dispersions will find diverse applications in tissue engineering, drug delivery, biosensing, stimuli and neural recordings. Today, the advantages of PEDOT:biopolymer dispersions are mainly given in terms of biocompatibility and the available studies show their potential to offer functional support and protection in oxidative environments when implanted in vivo or applied in vitro.

## 4. Applications of Innovative PEDOT-Based Materials

Conjugated polymeric materials can play an important role coupling conventional electronics with biology. These polymers and their derivatives have been widely investigated for applications in bioelectronics and biomedical devices. Although the commercially available PEDOT:PSS has already shown considerable potential for use in a variety of electronic medical devices, there are some important technical issues that limit their utility including their lack of (bio)functionality, relatively low resistance to fracture, biocompatibility, limited (bio)adhesion and lack of specific interactions with particular types of tissue [119].

**Figure 10:**
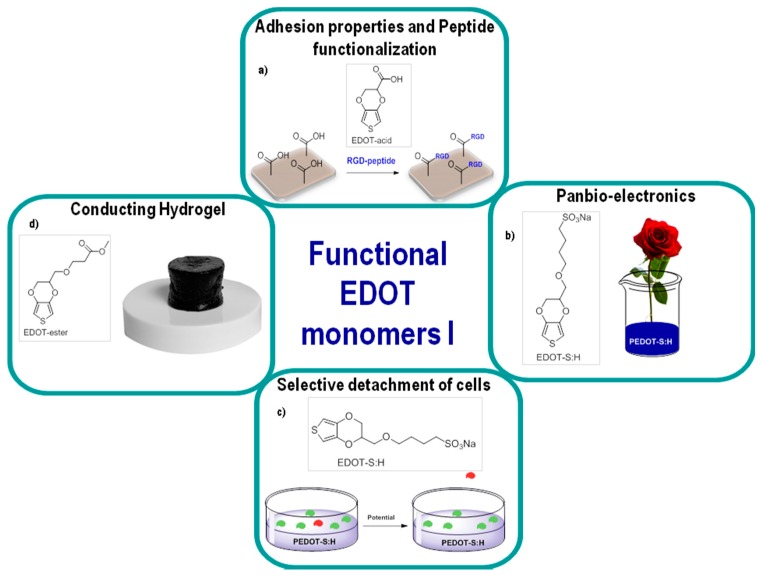
Different applications of functional EDOT monomers. (**a**) adhesion properties [120], (**b**) electronic-plants [121], (**c**) selective detachment of cells [122], (**d**) hydrogels [22].

However, to overcome these limitations, as was explained in Section 2 of this review, the chemistry of EDOT and ProDOT monomers can be systematically manipulated. Specific examples of this include an EDOT-acid monomer developed by Martin et al. which significantly improves the adhesion between PEDOT coating and metal substrates or transparent conducting oxide layers such as Indium tin oxide (ITO). EDOT-acid can be used to create PEDOT:PEDOT-acid co-polymers with systematic variations in surface wetting properties. An added value of the EDOT-acid monomer is that the acid groups make it possible to readily attach various biomolecules, such as peptides, onto the PEDOT co-polymer films. For instance, Povlich et al. used this method to create arginylglycyl-aspartic acid (RGD)-peptide-functionalized PEDOT films (Figure 10a). This work demonstrated that RGD-functionalized PEDOT films promoted adhesion and cell differentiation of primary rat motor neurons [13,120]. EDOT monomers derived from methyl alcohol functionalization (EDOT-OH), including carboxylic acids, acetates, azides and *N*-hydroxysuccinimide, could all be readily deposited on surfaces using electropolymerization from aqueous microemulsions [123]. 

An interesting self-doped and water-soluble derivative is PEDOT-sulfonate (PEDOT-S), developed by Grabielsson et al. [122,124]. PEDOT-S was utilized to introduce electronic functionality into plants [121]. In this work, they immersed a garden rose stem into a PEDOT-S aqueous solution forming a homogeneous and long-range hydrogel conductor phase along a plant tissue circuit (Figure 10b). Applying a potential to PEDOT-S films immersed in an electrolyte solution resulted in cell detachment and cell sorting (Figure 10c). The detachment is caused mainly by swelling due to the intake of charge-compensating ions and supporting electrolytes, and any cells cultured on top will detach along with the film.

Another example includes the ester, carboxylic acid and methacrylamide monomers developed by Marwad et al. [22]. These monomers are employed in the fabrication of electroactive hydrogels with the following characteristics: notable swelling ratio, good mechanical properties, electroactivity in physiological conditions, and suitability for proliferation and differentiation of C_2_C_12_ cells (Figure 10d).

**Figure 11:**
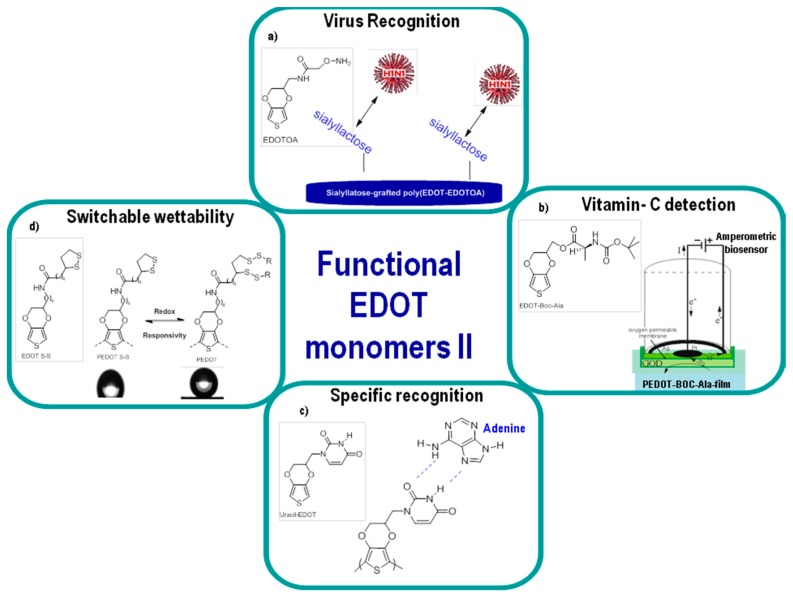
Different applications of functional EDOT monomers II. (**a**) virus recognition [125], (**b**) vitamin C detection [32], (**c**) specific recognition [47], (**d**) switchable wettability [126].

The biofunctionalized EDOT derivative bearing an oxylamine moiety (EDOTOA) was used to introduce trisaccharides, allowing the detection of the human influenza A virus (H1N1) (Figure 11a). For this purpose, EDOTOA was electrochemically co-polymerized with EDOT under optimized conditions. Then, sialyllactose trisaccharide was chemically introduced to the side chain of the electrodeposited conducting co-polymer films by glycosylation as the virus recognition element [125]. Specific interaction of sialyllactose with the human influenza A virus (H1N1) was detected by a quartz crystal microbalance.

A novel chiral l-alanine-modified 3,4-ethylenedioxythiophene (EDOT) precursor was synthesized by Lu et al. (Figure 11b). The corresponding polymer was used to fabricate an amperometric biosensor to determine the concentration of vitamin C [32]. Moreover, EDOT was covalently linked to the nucleobase uracil to obtain an uracil-EDOT monomer (Figure 11c). The corresponding conducting polymer obtained by the electropolymerization of EDOT-uracil was used as a sensor due to its specific recognition of the complementary base adenine [47]. Finally, Godeau and co-workers introduced a disulfide S–S bond on PEDOT in order to obtain a conducting polymer with reversible wetting properties (Figure 11d). In this work, based on a poly(3,4-ethylenedioxythiophene) bearing dithiolane groups, this strategy allowed for the reversible functionalization of surfaces with various thiol compounds [126]. A series of fluorinated PEDOT polymers which led to superhydrophobic surfaces was also developed using similar chemistry.

Some other examples not illustrated include the functionalized ProDOT monomers containing alkene side chains which made it possible to perform highly efficient click reactions when exposed to thiols in the presence of ultraviolet (UV) radiation. The functionalized PProDOTs created in this manner display huge differences in charge transport performance depending on whether the thiol group used was a charge-blocking alkyl group, a hydrophilic alkoxy moiety, or an electroactive ferrocene [79]. Furthermore, recently, Mantione et al. have reported PProDOT derivatives bearing bioactive dopamine (DA), TEMPO and tetraethylene glycol (TEG) functionalities [100].

**Figure 12:**
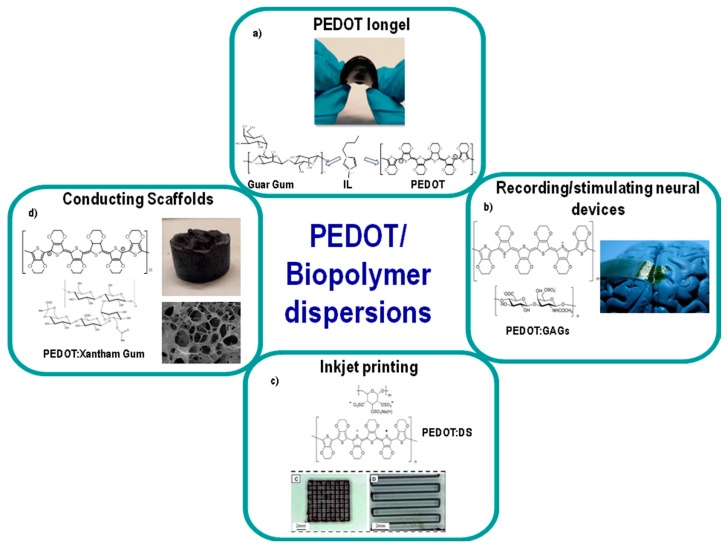
Different applications of PEDOT:biopolymer dispersions. (**a**) PEDOT ion gel [115], (**b**) recording/stimulating devices [113], (**c**) inkjet printing [112], (**d**) preparation of scaffolds [127].

A different family of new materials for bioelectronics are PEDOT:biopolymer dispersions. In these materials, the biopolymers substitute PSS and they are thoroughly mixed with PEDOT. These PEDOT:biopolymer dispersions are being used in different applications of bioelectronics as will be described next and illustrated in Figure 12.

The first application includes the development of conductive hydrogels and ion gels. Hydrogels containing polydioxythiophenes may also play an important role in tissue engineering. They improve small molecule transport, the degree of hydration and the mechanical flexibility of the material [128,129]. As a result, these conjugated polymer blends become even more similar to the extracellular matrix and have been used in various applications such as biosensors and drug delivery. The most intrinsic characteristic of conducting polymer hydrogels is the combination in the same material of the electroactivity given by the conducting polymer and the ionic conductivity given by the aqueous media. One drawback of conducting polymer hydrogels is that they ultimately dry due to water evaporation, losing their mechanical and electrical properties. In order to overcome the poor stability of the hydrogels, recently the first conductive polymer gel was prepared by a natural polysaccharide (guar gum), the conductive polymer PEDOT and an ionic liquid (IL). This material presents a unique combination of properties by mixing the electronic conductivity of PEDOT, the ionic conductivity and negligible vapor pressure of the ionic liquid and the mechanical softness and flexibility given by the polysaccharide [115]. Thus, conducting polymer gels can provide a mechanical buffer layer between the rigid electrodes and living tissues (Figure 12a).

Natural Glycosaminoglycans (GAGs) such as hyaluronic acid, heparin and chondroitin sulfate can also be combined with PEDOT. These PEDOT:GAG composites show full biocompatibility and a pronounced anti-inflammatory effect [113]. This last characteristic becomes crucial for in vivo applications or applications where there is a direct contact with cells (Figure 12b).

PEDOT:biopolymer dispersions composed by PEDOT and the sulfonated polysaccharide polyanion dextran sulfate (PEDOT:DS) have also been prepared. Water dispersions of this material can be successfully processed by drop-casting, spray-coating, inkjet printing and extrusion printing (Figure 12c). Furthermore, laser etching of dried films allows the creation of patterns with excellent definition, useful for the production of microscale devices [112].

The last example includes the development of porous conductive scaffolds based on PEDOT:biopolymer materials. These scaffolds are ideal for neuronal cell growth due to their conductive nature and the good compatibility between the polysaccharides and the cells [127] (Figure 12d).

## 5. Conclusions

In summary, the polydioxythiophenes PEDOT, and more recently ProDOT and their derivatives, have several attractive properties that include good stability, sufficiently high electrical conductivity and the ability to entrap and release biomolecules. They have shown tremendous potential in the field of organic bioelectronics, both in the domain of biosensing and also for integration with living cells (both in vitro and in vivo). Although the commercially available PEDOT:PSS has shown great promise for bioelectronics, in order to broaden the spectrum of applications, a functionalization of the monomers or the incorporation of biological active dopants is essential.

In the first section, we summarized the different methods that have been used to functionalize EDOT and ProDOT monomers, and also the different polymerization techniques that can be used to prepare the corresponding polymers. In the second section, we showed different biopolymers that can be employed to stabilize PEDOT and to improve its biocompatibility and cytotoxicity. In the third section, we summarized the applications of innovative PEDOT-type materials, including their use in biocompatible conductive polymer layers, conductive hydrogels, biosensors, selective detachment of cells, scaffolds for tissue engineering, electrodes for electrophysiology, implantable electrodes, stimulation of neuronal cells or pan-bio electronics.

However, the current state of biologically interfacing conjugated polymers requires further investigation into the long-term environmental stability and mechanical durability of conducting polymers. In fact, potential applications for bioelectronic devices, such as high-resolution neural recording of the brain, and 24 h monitoring of metabolite and disease-marker concentrations in the blood, will generate complex data, which must be analyzed in the long-term to determine their biological meaning. Furthermore, the diversity and synthetic versatility of conjugated polymers are expected to allow features such as biodegradability and printability, while maintaining the benefits associated with their softness and flexibility [130].

## Figures and Tables

**Table 1:** Available PEDOT:biomolecule dispersions prepared by chemical oxidative polymerization. Achieved conductivities, biocompatibility test, particle size, morphology and applications.

PEDOT:biomolecule	Conductivity (S·cm^−1^)	Application	Biocompatibility Test	Particle Size and Morphology
PEDOT:dextran sulfate [112]	7	Drug delivery, electrostimulation of cells	Cytotoxicity, L-929 cells	394–691 nm
PEDOT:DNA [110]	1	biosensing, drug delivery	-	50 nm fibers
PEDOT:heparin [113]	0.05–0.001	Recording/stimulating in vivo	Cytotoxicity, L-929 Cellular attachment, CCF-STTG1 cells Proliferation of SH-SY5Y Cells	>1 μm spheres
PEDOT:chondroitin Sulfate [113]	0.075–0.002	Recording/stimulating in vivo	Cytotoxicity, L-929 Cellular attachment, CCF-STTG1 cells Proliferation of SH-SY5Y Cells	500 nm spheres
PEDOT:hyaluronic acid [113]	0.071–0.003	Recording/stimulating in vivo	Cytotoxicity, L-929 Cellular attachment, CCF-STTG1 cells Proliferation of SH-SY5Y Cells	200 nm spheres
PEDOT:sulfated cellulose [111]	0.576	-	-	250–350 nm
PEDOT:pectin [114]	˂0.01	-	-	-
PEDOT:guar gum [115]	0.028–0.129	Iongels	-	100–300 nm spheres
